# Individual Radiation Exposure Dose Due to Support Activities at Safe Shelters in Fukushima Prefecture

**DOI:** 10.1371/journal.pone.0027761

**Published:** 2011-11-16

**Authors:** Satoru Monzen, Masahiro Hosoda, Shinji Tokonami, Minoru Osanai, Hironori Yoshino, Yoichiro Hosokawa, Mitsuaki A. Yoshida, Masatoshi Yamada, Yasushi Asari, Kei Satoh, Ikuo Kashiwakura

**Affiliations:** 1 Department of Radiological Life Sciences, Hirosaki University Graduate School of Health Sciences, Hirosaki, Japan; 2 Hirosaki University Research Institute for Radiation Emergency Medicine, Hirosaki, Japan; 3 Department of Emergency and Disaster Medicine, Hirosaki University Graduate School of Medicine, Hirosaki, Japan; East Carolina University, United States of America

## Abstract

Immediately after the accidents in the nuclear power stations in Fukushima on March 11, the Japanese Government ordered the evacuation of the residents within a 20-km radius from the station on March 12, and asked various institutions to monitor the contamination levels of the residents. Hirosaki University, which is located 355 km north of Fukushima City, decided to send support staff to Fukushima. This report summarizes the results of the exposure of 13 individual teams from March 15 to June 20. The support teams surveyed more than 5,000 people during this period. Almost all subjects had external contamination levels of less than 13 kcpm on Geiger-Müller (GM) survey meter, which is categorized as “no contamination level.” The 1^st^ team showed the highest external exposure dose, but the 4^th^ team onward showed no significant change. Subsequently, the internal radiation exposure was measured using a whole body counter that indicated undetectable levels in all staff members. Although the measured external radiation exposure dose cannot have serious biological effects on the health of an individual, a follow-up study of the residents in Fukushima and other regions where the radioactive material has spread will be required for a long time.

## Introduction

On March 11, an earthquake measuring 9.0 on the Richter scale and a subsequent tsunami caused extensive damage to the Fukushima Dai-ichi Nuclear Power Station operated by the Tokyo Electric Power Company. Subsequently, this series of events caused a phreatic explosion in unit 1 on March 12, at 15:36; then in unit 3 on March 14, at 11:01; and finally in units 2 and 4 on March 15 at around 9:00. A large amount of radioactive materials were released into the environment. On March 12, the Japanese Government ordered the evacuation of the residents who lived in an area within 20 km from the nuclear power station.

Hirosaki University, just after the first phreatic explosion, sent many of its staff members to the safe shelters that were set temporarily for the residents of the area around the nuclear power station, to support the radiation survey and monitor the contamination of the safe shelters. Immediately after the accident, there was no accurate information regarding the radiation/radioactivity levels in the area of the nuclear power station, because the conditions of each nuclear power station were unstable. In addition, due to the confusion in the north of Honshu, which is the main island in Japan, the means of transportation were limited, and it was very difficult to obtain gas for vehicles as well as dry cell batteries for survey radiation meters and other types of equipment. The highway that connects Hirosaki to Fukushima was also closed, and only emergency vehicles with permission of the Government could use it. During our stay in the area, all members of the Hirosaki University team installed personal dosimeters. Although some reports showed the extent of the accidents in the Fukushima Dai-ichi Nuclear Power Station [1]–[6], no report on the support activities at Fukushima, especially during the early stages of the accident, had been disclosed. This report summarizes the exposure results of the 13 individual teams from March 15 to June 20. Continued support is being provided. In addition, the dose rate in air was measured during the trip from Hirosaki City to Fukushima City.

## Results and Discussion

**Figure 1:**
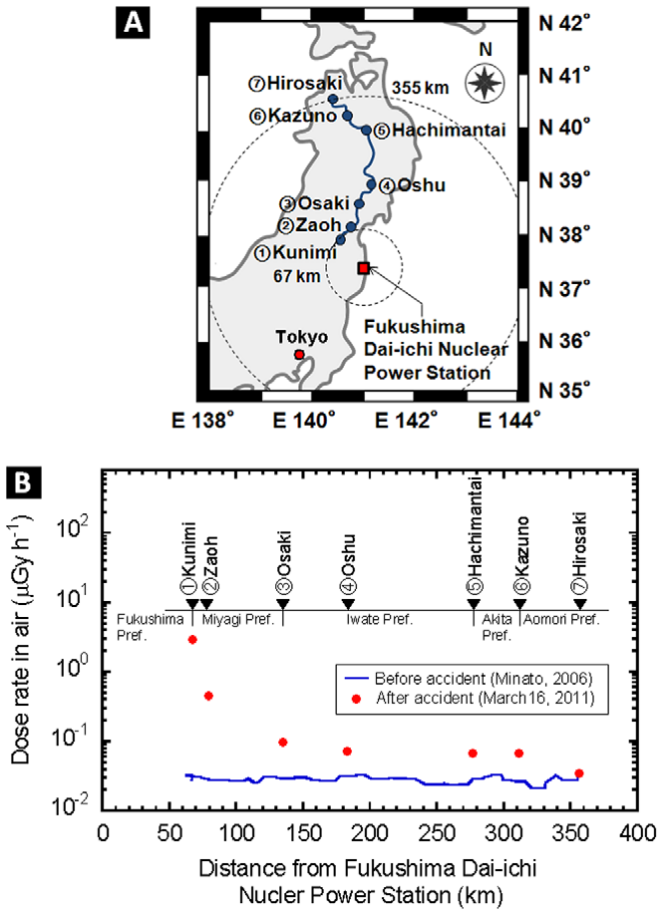
Location of Hirosaki City, Fukushima City and Fukushima Dai-ichi Nuclear Power Station on the map of eastern Japan (A). The results of the absorbed dose rate in air at the seven parking area during the move from Hirosaki City to Fukushima City. The line shows the data measured at 2006 by Minato [8] (B).

**Figure 2:**
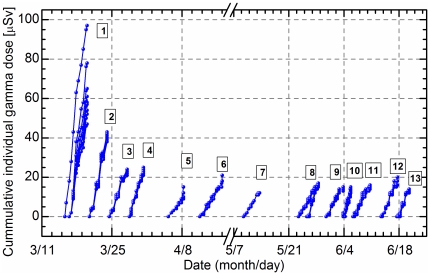
Cumulative individual radiation dose accumulated daily. The survey teams have started measuring our departure from Hirosaki.

The 1^st^ team measured the absorbed dose rate in air outdoors at the seven parking areas during the move from Hirosaki City to Fukushima City on March 16. As shown in Figure 1, the dose rate gradually increased from Oshu City, which is located about 180 km from Fukushima Dai-ichi Nuclear Power Station, to a value of 10 µGy h^−1^ at midtown of Fukushima city. The dose rates in air from artificial radionuclide were calculated using analysis software developed by Minato [7]. The contributions of Te-132, I-131, Cs-137, Cs-134, and I-132 show increased values when approaching the Fukushima Dai-ichi Nuclear Power Station from Osaki, Miyagi Pref.; however, the contributions of the aforementioned isotopes are hardly detected in Hirosaki city (Table 1). Minato had reported the dose rate in air around our measurement point in 2006 [8]. The values in Figure 1, which are shown as a line, indicate no difference between the routes and no contamination level. From March 15 to June 20, Hirosaki University had sent more than 100 of its staff members to various locations in Fukushima Prefecture following a request by the Government. They surveyed more than 5,000 people during this period. The details are as follows: the 1^st^ team surveyed 928; the 2^nd^ team surveyed 486; the 3^rd^ team surveyed 655; the 4^th^ team surveyed 1400; and the each of 5-13^th^ team surveyed about 100 to 700 people, respectively. The 1-4^th^ team was put in charge of the logistic activities in the disaster area conducted by Fukushima University (no data). Ten residents in total showed high values, which were, however, less than 100 kcpm, and they required no decontamination. Almost all of the surveyed individuals had external contamination levels of <13 kcpm. The absorbed dose rate outdoors in the areas of the safety shelters was independent of the distance from the nuclear power station; its value was quite different for each individual area, which suggests that environmental pollution by radioactive material had occurred early. The dose rate values at each location gradually reduced over time. The values of the absorbed dose rate in the indoor air were less than one-tenth of those in the outdoor air. The external exposure dose of the staff showed the highest value in the 1st team, after which the value decreased; finally, no change was observed after the 3^rd^ team (Figure 2). Subsequently, the internal radiation exposure of each staff member was measured using a whole body counter at Hirosaki University. The results showed undetectable levels in all staff members (data not shown).

**Table 1:** Contribution of radionuclide to dose rate estimated on expressway from Fukushima Prefecture to Aomori Prifecture.

No.	DateTime	Site	Longitude	Latitude	Distance from FNPS* (km)	Natural radiation	Artificial radiation	Dose rate in air (μGy h^−1^)					
						(μGy h^−1^)	(μGy h^−1^)	Te-132	I-131	Cs-137	Cs-134	I-132	Cs-136
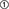	2011/3/1916:37	Kunimi, Fukushima Pref.(Kunimi)	N37.907°	E140.577°	67	0.032	2.897	0.203	0.116	0.087	0.956	1.506	0.029
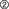	2011/3/1917:16	Zaoh, Miyagi Pref.(Zaoh)	N38.070°	E140.660°	79	0.029	0.421	0.029	0.034	0.017	0.109	0.231	0
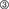	2011/3/1919:03	Osaki, Miyagi Pref.(Chojahara)	N38.637°	E140.959°	135	0.029	0.067	0.005	0.007	0.002	0.016	0.037	0
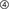	2011/3/1921:08	Oshu, Iwate Pref.(Maesawa)	N39.067°	E141.101°	183	0.032	0.040	0.003	0.003	0.002	0.009	0.023	0
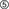	2011/3/1922:59	Hachimantai, Iwate Pref.(Iwatesan)	N39.914°	E141.046°	277	0.029	0.039	0.004	0.002	0	0.004	0.029	0
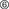	2011/3/200:09	Kazuno, Akita Pref.(Hanawa)	N40.187°	E140.803°	312	0.026	0.039	0.004	0.002	0	0	0.033	0
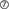	2011/3/201:55	Hirosaki, Aomori Pref.(Hirosaki Univ.)	N40.601°	E140.467°	355	0.031	0.003	0	0	0	0	0.002	0

*FNPS: Fukshima Dai-ichi Nuclear Power Station.

A follow-up study of the residents of Fukushima and other regions where the radioactive material has spread will be required for a long time in the future. Presently, we have been analyzing the various environmental samples collected at many different locations, especially the northwest regions of Fukushima Prefecture, e.g., Kawamata Town, Namie Town, and Iitate Village. There are many “hot spot areas” where radioactivity has accumulated locally. We have to continue the various measurements of the absorbed dose rate in air and the state of radioactivity. These measurements can certainly help to estimate the cumulative effect of low-dose radiation in humans, and will be required to determine short- and long-term health effects such as carcinogenesis or leukemia, especially in fetuses and infants. The serious accidents that occurred in the Fukushima Dai-ichi Nuclear Power Stations shocked the world, and they have and will still affect our society. The Japanese people have a responsibility to continue research on the effect of radioactivity in humans.

## Materials and Methods

### Planning for support activities

The incidents that occurred in the Fukushima Dai-ichi Nuclear Power Station are presented in Table 2 as time series data. On March 12 during the failure of the electric power supply in the city of Hirosaki, the president of Hirosaki University, Dr. Endo Masahiko, called an emergency meeting of the Hirosaki University Radiation Safety Organization to discuss the dispatch of support to Fukushima following a request from the Ministry of Education, Culture, Sports, Science & Technology of Japan. The plan for the support activity was discussed and decided in this meeting. Each team consisted of 3–10 members, which included medical doctor, nurses, a radiological technologist or staff for radiation measurement, and an office employee.

**Table 2:** The incidents occurred in the Fukushima Dai-ichi nuclear power station.

Date, time	Incidents	Response
March 11, 2011,	14:46; 9.0-Magnitude Earthquake and power failure in eastern Japan.	
	15:30; Fukushima Nuclear Power Station stop.	
	15:46; Tsunami hits the Fukushima Dai-ichi nuclear power stations a few times (about 45-50 feet above sea level and about 13-17 feet inundation above the floor level).	21:30; Evacuation directive to residents who live within 3 km from nuclear power stations.
March 12	15:36; Hydrogen explosion at unit 1.	18:25; Evacuation directive to residents who live within 20 km from nuclear power stations.
March 13		9:00; Preparation of dispatch unit in Hirosaki university.
March 14	11:01; Hydrogen explosion at unit 3.	
March 15	06:00; An abnormal noise began emanating from nearby pressure suppression chamber and the pressure within the chamber decreased at unit. TEPCO^#^ confirmed the explosive sound and the sustained damage around the 5th floor rooftop area of the nuclear reactor building at unit 4.	11:00; The first support team of Hirosaki University depart for Fukushima.
April 08		Electric power restoration in east Japan.

### Measuring instruments

The cumulative external radiation was estimated using a personal dosimeter (PDM-112, Hitachi-Aloka Medical, Ltd., Mitaka, Tokyo, Japan) (γ(X)-ray type, 40 keV∼, 1 µSv-10 mSv). For the analysis of the absorbed dose rate in air, a scintillation spectrometer was used (JSM-112, Hitachi-Aloka Medical, Ltd., Mitaka, Tokyo, Japan) (γ rays, 30 keV–5 MeV, ϕ76.2 × 76.2 mm NaI(Tl), Dose rate  =  BG-10 µGy h^−1^). GM survey meter were used for the purpose of individual survey (TGS-146, Hitachi-Aloka Medical, Ltd.) (β(γ)-ray type, 0–99.9 kcpm, ϕ50 mm, 19.6 cm^2^, Window thickness: 2.5 mg cm^−2^). The internal radiation exposure of each staff member was measured by a whole body counter (Hitachi-Aloka Medical, Ltd., Mitaka, Tokyo, Japan).

### Measurement of the radiation exposure dose

Hirosaki University is located in Hirosaki in Aomori Prefecture and is located 355 km north from Fukushima City. During the move from Hirosaki City to Fukushima City, the absorbed dose rate in air on the pavement was measured and recorded at seven parking areas, the midtown of Fukushima City, and the place of the support activities. In our previous study, we reported that the dose rates on the unsealed surfaces were 1.0–2.1 times (average: 1.3 times) higher than those on the pavement [9]. This finding implies that contamination on the pavement can be removed due to washout by rainfall and re-entrainment by wind [10]–[13]. We reported that the dose rates on the unsealed surfaces were from 1.0 to 2.1 times (average: 1.3 times) higher than those on pavement [9]. That is, the contamination on pavement can be removed with washout by rainfall, and re-entrainment by wind [10]–[13]. Measurement of the radiation exposure of each individual by a personal dosimeter was performed starting from the beginning of the trip at Hirosaki up to the return to Hirosaki.

### Outline of the support activity in Fukushima Prefecture

The 1st team left on March 15. The support activity started with a conference in the Headquarters from 08:00 to about 18:00. After the support activities of the day, all staff members attended a conference at 19:00 or 20:00. Almost staff members performed screening of the external exposure of the residents inside each shelter. The activity area was located 20 km outside the Fukushima Dai-ichi nuclear power station in Fukushima and Miyagi Prefecture. The results of each individual survey were grouped into three categories as follows: less than 13,000 cpm on GM survey meter is no contamination, a value between 13 kcpm and 100 kcpm is of no particular concern and action, and more than 100 kcpm is contamination that requires decontamination.
